# Challenges in predicting future high-cost patients for care management interventions

**DOI:** 10.1186/s12913-023-09957-9

**Published:** 2023-09-14

**Authors:** Chris Crowley, Jennifer Perloff, Amy Stuck, Robert Mechanic

**Affiliations:** 1 West Health Institute, 10350 N Torrey Pines Rd, La Jolla, CA 92037 USA; 2 Institute for Accountable Care and Brandeis University, 415 South St. MS 035, Waltham, MA 02453 USA

**Keywords:** Medicare, High-cost patients, Utilization, Care management, Risk-stratification, Segmentation

## Abstract

**Background:**

To test the accuracy of a segmentation approach using claims data to predict Medicare beneficiaries most likely to be hospitalized in a subsequent year.

**Methods:**

This article uses a 100-percent sample of Medicare beneficiaries from 2017 to 2018. This analysis is designed to illustrate the actuarial limitations of person-centered risk segmentation by looking at the number and rate of hospitalizations for progressively narrower segments of heart failure patients and a national fee-for-service comparison group. Cohorts are defined using 2017 data and then 2018 hospitalization rates are shown graphically.

**Results:**

As the segments get narrower, the 2018 hospitalization rates increased, but the percentage of total Medicare FFS hospitalizations accounted for went down. In all three segments and the total Medicare FFS population, more than half of all patients did not have a hospitalization in 2018.

**Conclusions:**

With the difficulty of identifying future high utilizing beneficiaries, health systems should consider the addition of clinician input and ‘light touch’ monitoring activities to improve the prediction of high-need, high-cost cohorts. It may also be beneficial to develop systemic strategies to manage utilization and steer beneficiaries to efficient providers rather than targeting individual patients.

## Background

Chronic disease accounts for most US healthcare spending [1]. Hospitalizations associated with chronic diseases are expensive and increase risk of harm to frail older patients [2]. Within Medicare value-based payment models, providers are incentivized to proactively engage patients and coordinate care to reduce high-cost service utilization such as hospitalizations. Given the investments required to operate care management programs, value-based providers, including accountable care organizations (ACOs), need to allocate resources as efficiently as possible, targeting resources towards patients who are the most likely to benefit. This includes efforts to triage and treat beneficiaries in the community to avoid hospitalization and to provide transitional support for those who have an admission. Currently, organizations use some combination of predictive algorithms, historical utilization and clinician referral to identify patients for care management [3]. However, little is known about the efficiency or efficacy of these identification processes [4–6]. Moreover, specific mechanisms contributing to possible inefficiencies in targeting resources to high-need, high-cost beneficiaries have not been identified or characterized in depth. We identified and investigated two such mechanisms arising in a heart failure segmentation algorithm used to identify Medicare beneficiaries who are more likely to have a hospital admission.

Despite the widespread adoption of risk stratification, there is limited evidence that care management can be targeted in a manner that consistently lowers net spending [7–9]. Several studies suggest that Medicare ACO savings do not appear concentrated among patients with high or complex needs [10, 11]. What’s more, historically under-served populations are less likely to be identified by data driven algorithms that rely on coding in administrative data [12]. In order to investigate possible factors contributing to these results, we hypothesized two underlying mechanisms of inefficiency that may arise when risk-stratifying Medicare beneficiaries: (1) narrow cohort focus; and (2) utilization heterogeneity.

**Narrow cohort focus**. In forming our hypothesis, we first noted that progressively more restrictive segments should more successfully predict future high utilizing patients. However, because the more restrictive segments contain commensurately fewer beneficiaries, they represent less of the total hospital utilization in a covered population. If care management is only targeted to particular segments, opportunities to reduce hospital utilization for beneficiaries outside of the segments may be foregone.

**Utilization heterogeneity**. The second source of inefficiency arises because even within progressively more restrictive patient segments, there is still substantial heterogeneity in performance year hospital use. This means that within targeted cohorts a small number of beneficiaries may accumulate a considerable number of hospitalizations in the subsequent year, while a sizable proportion of the same cohort will not be hospitalized at all. Resources devoted to reducing hospitalizations for beneficiaries who would not have been hospitalized anyway may provide other benefits, but from a financial perspective the activity is unlikely to generate a favorable return on the investment.

This study used a heart failure segmentation algorithm. Heart failure is significant because it is highly prevalent in the Medicare population and because per-beneficiary spending for patients with heart failure is approximately twice the Medicare average [13, 14]. Moreover, there are disparities in care access and heart failure outcomes between African American, Hispanic and White beneficiaries, making any investigation of heart failure segmentation a potential avenue to address health equity. Specifically, development of more objective criteria for allocation of resources could overcome both conscious and unconscious bias in decisions related assignment of care managers to patients that might benefit. We specifically investigated inpatient hospital utilization for three increasingly narrow cohorts of Medicare heart failure patients defined using base year (2017) utilization. We then measured utilization in the subsequent performance year (2018) as well as total per-beneficiary, per-year spending. Finally, within each segment, we examined the proportion of beneficiaries with zero 2018 inpatient admissions, for whom care management may have potentially limited impact on total spending.

## Methods

This observational cohort study was based on a 100% sample of Medicare claims and demographic data contained in a two-year window spanning 2017 and 2018. In the United States, Medicare provides health insurance for seniors aged 65 and over, with Medicare Part A covering hospital care, Part B covering outpatient care, and Part C (Medicare Advantage) representing the same coverage as Parts A and B but through an alternative commercial offering. The study population was Medicare beneficiaries eligible for attribution to an ACO (i.e., at least one qualifying Evaluation and Management (E&M) services from an ACO physician in 2017). To identify this group, we start with Medicare beneficiaries who were continuously enrolled in Medicare Parts A and B for the whole study period (2017–2018). We excluded beneficiaries with end-stage renal disease (ESRD), in part because ESRD patients are atypical from the rest of the heart disease cohort and would need to be assessed separately. Moreover, ESRD patients are often attributed to specialized ACO models, also necessitating separate analysis. We also excluded beneficiaries who died in 2017 and beneficiaries who enrolled in a Medicare Advantage plan (Part C) at any time in 2017 or 2018. We retained patients that died in 2018, provided they had continuous enrollment in Medicare Parts A and B prior to death. We treated 2017 as a base year in which we applied three progressively restrictive definitions of heart failure to create three cohorts within the study population. We then produced descriptive statistics to compare the performance year in the three heart failure cohorts using the overall study population described above as a basis for comparison. The three heart failure cohorts were defined as follow:

### Segment 1

Beneficiaries with at least **one** 2017 ambulatory (Part B) bill or a 2017 index inpatient (Part A) admission with a heart failure ICD-10 diagnosis code (N = 3.6 million).

### Segment 2

Beneficiaries with at least **two** 2017 ambulatory (Part B) heart failure bills 30-days apart or one 2017 index hospitalization with a primary diagnosis of heart failure (N = 1.9 million).

### Segment 3

Beneficiaries with one or more 2017 index hospitalizations with a primary diagnosis of heart failure (N = 1.0 million).

Each of the more restrictive segments was also contained within the less restrictive segments, allowing the narrowing effects of the heart failure segmentation algorithm to be observed. It is important to note that we could have investigated alternative base-year inclusion criteria such as the top 5% or 1% spenders, or those that had zero hospitalizations in the base year.

### Measures

Outcome measures include 2018 performance year all-cause, acute-care hospitalizations per thousand beneficiaries. Admissions were identified using Medicare Part A bills with a type code of 60, indicating an acute-care stay. We also calculated the percentage of total study population hospitalizations in 2018, and the percentage of beneficiaries with zero 2018 hospitalizations per segment. To assess total cost of care, we calculated total per-beneficiary, per-year (PBPY) spending as the sum of all Medicare Part A and B Medicare reimbursements for both 2017 and 2018.

### Analysis

We used the 2017 claims data to establish both the overall study population as well as the cohorts included in the segments described above. We then used the 2018 claims data to determine 2018 outcome measures for each segment. We plotted utilization histograms showing the distribution of 2018 hospitalization counts across the three segments. All distributions as well as the outcome measures were compared with corresponding variables from the overall ACO-attributable Medicare population. This study was reviewed by the Brandeis University IRB and deemed to be an exempt study.

## Results

The study population consisted of approximately 26 million beneficiaries. As shown in Table 1, the least restrictive heart failure segment (Segment 1) yielded a cohort containing about 3.6 million beneficiaries while the more restrictive segments yielded about 1.8 million and 1.0 million beneficiaries for Segments 2 and 3 respectively. In general, the cohorts were similar in terms of reasons for eligibility, but the 2018 mortality rate was higher for the narrowest cohort (21% for cohort 3) compared to the most inclusive cohort (13% for cohort 1). Average annual 2017 Medicare spending for the narrowest cohort was $46,440 compared with $22,947 for the most inclusive cohort. Performance year spending was generally lower, with 2018 spending averaging $27,037 for the narrowest cohort compared with $18,391 for the most inclusive cohort.

**Table 1:** Characteristics of Heart Failure Cohorts in 2017

	Segment 1	Segment 2	Segment 3
Number	3,639,259	1,897,108	1,025,745
Mean Age	77	78	77
Sex (Female)	54%	53%	54%
Eligibility Category			
-Disabled (%)	25%	25%	26%
-Aged dually eligible for Medicaid and Medicare (%)	14%	15%	15%
-Aged, non-dual (%)	61%	60%	58%
ACO Status (%)	31%	31%	32%
Mortality 2018 (%)	13%	17%	21%
Mean PMPY 2017 (Truncated)	$22,947 ($29,444)	$30,968 ($34,255)	$46,440 ($35,467)
Mean PMPY 2018 (Truncated) (STD)	$18,391 ($27,335)	$21,689 ($30,495)	$27,037 ($35,322)
Zero Admissions 2018 (%)	67.5%	62.2%	53.6%

**Segment 1**: Beneficiaries with at least one ambulatory (Part B) bill or an inpatient (Part A) with a heart failure ICD-10 diagnosis code

**Segment 2**: Beneficiaries with at least two ambulatory (Part B) heart failure bills 30-days apart or one hospitalization with a primary diagnosis of heart failure

**Segment 3**: Beneficiaries with one or more hospitalization with a primary diagnosis of heart failure

Table 2 shows the 2018 hospital admissions for each heart failure cohort and for the overall study population (labeled All FFS beneficiaries). The study population comprised of 26 million beneficiaries accounted for about 6 million hospitalizations in 2018. The three progressively more restrictive segments contained commensurately smaller proportions of the total hospitalizations. Segment 1, the least restrictive segment, contained about 1.9 million or 30% of the 6 million hospitalizations. Segments 2 and 3 contained about 1.2 million (19%) and 0.8 million (13%) respectively of the 6 million hospitalizations in the study population. Table 2 indicates the degree to which the hospitalization rates are dramatically higher. For example, hospitalization rates in Segment 1 are twice the national average of 240 admissions per 1,000 beneficiaries. Hospitalization rates are nearly 3 and 4 time higher than the national average in Segments 2 and 3 respectively. Finally, as shown in Tables 2, 84% of beneficiaries in the study sample had zero hospitalizations in 2018, compared with 68%, 62% and 54% respectively in the three heart failure cohorts.

**Table 2:** Beneficiary counts and outcome measures for all Medicare ACO Assignment-Eligible Beneficiaries and for Three Heart Failure Segments in 2018

2018 Beneficiaries from 2017 Cohort	Number of Beneficiaries	Total Hospital Stays	Hospital Stays per 1,000 Beneficiaries	Number of Beneficiaries with Zero Hospital Stays	Percent Zero Hospital Stays
**All Fee-for-service Beneficiaries**	25,587,927	6,138,763	240	21,531,217	84%
**Heart failure segment 1**	3,160,002	1,859,564	588	2,133,143	68%
**Heart failure segment 2**	1,580,272	1,157,220	732	982,843	62%
**Heart failure segment 3**	810,045	795,952	983	433,912	54%

**Segment 1**: Beneficiaries with at least one ambulatory (Part B) bill or an inpatient (Part A) with a heart failure ICD-10 diagnosis code

**Segment 2**: Beneficiaries with at least two ambulatory (Part B) heart failure bills 30-days apart or one hospitalization with a primary diagnosis of heart failure

**Segment 3**: Beneficiaries with one or more hospitalization with a primary diagnosis of heart failure

**Fig. 1:**
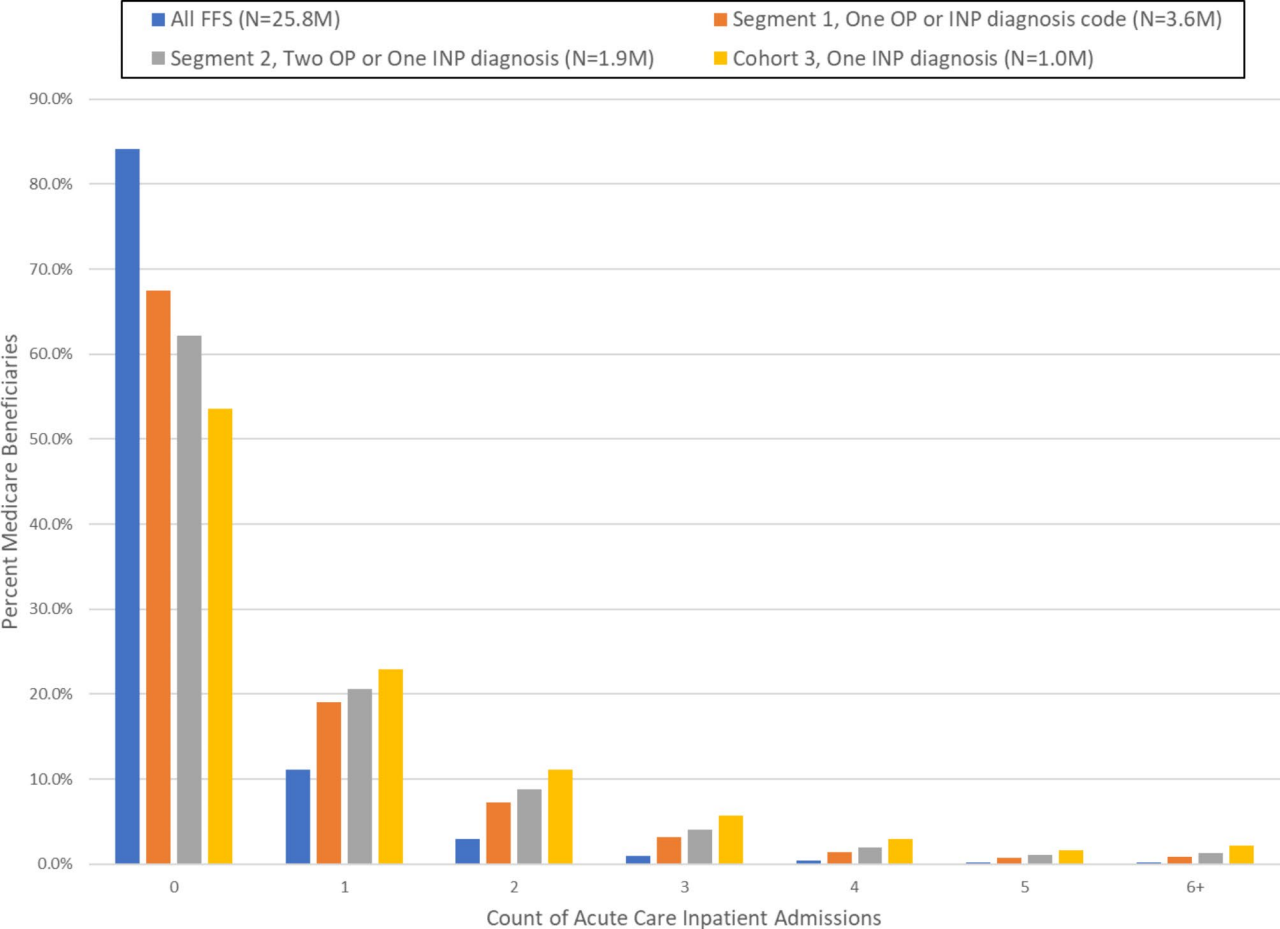
Distribution of Hospital Utilization Across Study Cohorts, Count of hospital admission (0–6 or more) by Heart Failure Cohort, 2018 Notes: **Segment 1**: Beneficiaries with at least **one** 2017 ambulatory (Part B) bill or a 2017 index inpatient (Part A) admission with a heart failure ICD-10 diagnosis code (N = 3.6 million) **Segment 2**: Beneficiaries with at least **two** 2017 ambulatory (Part B) heart failure bills 30-days apart or one 2017 index hospitalization with a primary diagnosis of heart failure (N = 1.9 million) **Segment 3**: Beneficiaries with one or more 2017 index hospitalizations with a primary diagnosis of heart failure (N = 1.0 million)

Figure 1 shows histograms of 2018 counts of hospital utilization for the overall study population and each heart failure cohort including the prevalence of zero 2018 admissions. Overall, the basic shape of the distribution is the same despite the fact the admissions rates are much higher for the progressively more restrictive segments.

## Discussion

Our study shows that increasingly restrictive heart failure segments do, in fact, isolate patients with dramatically higher rates of hospitalization—roughly 2, 3 and 4 times, respectively, compared to the overall study population. Thus, the restrictive segments may appear to provide viable targets for care management. However, as shown in Tables 1 and 2, less than one third of the hospitalizations from the overall population are represented in the largest segment, and that proportion drops to only 13% of total hospitalizations for the most restrictive segment. This illustrates the mechanism by which a narrow cohort focus misses substantial hospital utilization outside of the target group. By focusing on cohorts with higher likelihood of future hospitalizations, segmentation algorithms could exclude the majority of patients destined to be hospitalized in the performance year. Additionally, between half and two-thirds of beneficiaries in the 3 heart failure cohorts had zero hospitalizations in 2018. Thus, despite isolating cohorts with significantly higher hospitalization rates than the overall Medicare FFS population, the targeted beneficiary segments still have a high likelihood of not being hospitalized. This illustrates the mechanism of utilization heterogeneity. Assigning care managers to historically high-cost enrollees may provide significant patient benefits, [9] but our analysis suggests it may not be an efficient means for allocating resources towards reducing hospitalizations. What’s more, these strategies ignore historically underserved populations who are less likely to have a broad array of diagnoses in claims or electronic health record data, further perpetuating disparities.

Even though this study investigated only one, relatively simple approach to segmenting beneficiaries, the methodology may be applicable to other risk stratification strategies. The key considerations are the proportion of beneficiaries who may be ‘missed’ and the proportion who may receive care management support that would not have incurred a hospitalization in the performance year even without such support. The two mechanisms discussed here are both ubiquitous and unavoidable. We know from actuarial science literature that beneficiary risk pools exhibit spending distributions similar to the utilization patterns illustrated in this paper [15, 16]. In other words, targeting algorithms include some degree of error. This may be driven by missing information related to the beneficiary’s health status or the limitations in the statistical or data driven techniques used to identify the cohorts. This may also explain why clinician referral is frequently used as a compliment to data driven targeting methodologies. Analysis of clinician referral as a complement to the segmentation approaches is beyond the scope of the present study. However, given the challenges identified herein, future research may be directed toward quantifying the degree to which more real time referrals might remediate some of the challenges presently identified, paying special attention to personalized relationships between patients and providers that may not be captured in purely data-driven approaches.

### Limitations

This study has several important limitations. First, it was based on claims data which contain limited information about patient medical conditions. Second, it only examined one approach to segmenting Medicare beneficiaries with heart failure. As indicated earlier, we could also have examined yet more restrictive (narrower) cohorts, and could also have considered broader segments, including segments exhibiting overall traits of good health (e.g. zero base year hospitalizations). We also note that many who exhibited zero hospitalizations in the performance year may still have been at significant risk for hospitalization, with that risk carrying over to subsequent years. Stated differently, one year may not have been enough time for that risk to be manifest as hospitalizations. Moreover, it is possible that care coordination resources—even those provided during a performance year of zero hospitalizations—could ultimately benefit those patients in subsequent years. Third, our study used a 100% sample of Medicare beneficiaries. By excluding Medicare Advantage (MA) patients, we may be missing important variables associated with the resources already allocated through the supplement benefits that may be provided to MA patients. In practice, a provider adopting a value-based payment model would only see its own attributed lives, which may only approximate the underlying distribution. Finally, our approach assessed potential inefficiencies associated with beneficiary segmentation with respect to hospital admissions. However, care management also influences other types of utilization such as post-acute care.

Worth noting, the data supporting the findings of this study are available from the Center for Medicaid and Medicare Services (CMS) but restriction apply to the availability of these data, which were used under license for the current study and so are not publicly available. However, similar data are available upon request and with permission of CMS.

## Conclusions

If all risk stratification approaches are limited by the two mechanisms described in this paper, it implies that risk stratification alone cannot efficiently target care management resources. This suggests a need for risk-based providers to augment segmentation strategies with additional information from clinicians, family members, patient surveys or remote biometric monitoring [17]. Such information could support more adaptive, real-time decision making and deployment of resources such as paramedic or clinician home visits, rather than relying exclusively on a-priori base-year parameters to allocate care management resources. Although promising in concept, the few high-quality studies examining the impact of remote monitoring technologies on outcomes have shown mixed results [18]. The Centers for Medicare and Medicaid Services recently established coverage for remote patient monitoring (RPM) services with maximum annual payments of $1,460 per patient [19]. But at these payment levels, the use of RPM services may be subject to the same inefficiencies as traditional care coordination. Lower cost surveillance techniques may be necessary to support system-level changes.

ACOs and other provider groups need to consider systems-level solutions that promote efficient care for all beneficiaries, including those approaching, but not yet in, the late stages of chronic illness. Rather than focusing on individual high-cost patients, health care organizations could generate savings more effectively by implementing systematic initiatives to increase efficiencies – for example: implementing decision support systems to refer patients to efficient medical specialists, reducing capacity to perform overused procedures or establishing systems to shift care from hospital outpatient departments to physician offices when appropriate. However, strategies that target individual patients are more appealing to health systems that are predominantly fee-for-service. Systemic approaches to reduce spending may help them succeed in value-based contracts, but they will reduce their fee-for-service revenue [20].

## Data Availability

This analysis uses Medicare claims data from the Center for Medicaid and Medicare Services Virtual Data Research Center under an Innovator DUA. We are not able to share the person level analytic files given the constraints of our data use agreement. However, we are willing to share the analytic code upon request. Please direct request to Jennifer Perloff at perloff@brandeis.edu.

## Appendix: ICD-10 Codes used to Identify Heart Failure

**Heart failure algorithm:** Any diagnosis on the claims, One or two claims required depending on the algorithm.

**ICD-10 code:** I09.81, I11.0, I13.0, I50.1. I50.21, I50.22, I50.23, I50.31, I50.32, I50.33, I50.40, I50.41, I50.42 I50.43, I50.811, I50.812, I50.813, I50.814, I50.82, I50.83, I50.84, I50.89, I50.9.

## List of Abbreviations

ACOs: Accountable Care Organizations
E&M: Evaluation and Management
ESRD: End Stage Renal Disease
FFS: Fee for Service Medicare
MA: Medicare Advantage
PBPY: Per-beneficiary Per-year
RPM: Remote Patient Monitoring
